# Chicago Medical College, University Day

**Published:** 1886-04

**Authors:** 


### LOCAL NEWS.

RUSH MEDICAL COLLEGE.—The Annual Commencement of Rush Medical College was held on Tuesday, February 16. Diplomas were conferred on 156 graduates. Five diplomas were conferred *pro causa honoris.* The total number of matriculates during the winter session was 404; during the preceding spring term, 138.

Professor Norman Bridge, M.D., formerly Professor of Hygiene, and Adjunct Professor of the Principles and Practice of Medicine, has been elected Professor of General Pathology.

Hereafter, practical work in the physiological and pathological laboratories will be required of undergraduates.

THE COLLEGE OF PHYSICIANS AND SURGEONS OF CHICAGO.—The Annual Commencement of the College of Physicians and Surgeons of Chicago was held on Tuesday, February 23. Degrees were awarded to 71 members of the same class, which numbered 85. During the winter session there were 165 matriculates. Dr. E. F. Wells, of Minster, Ohio, has been elected Professor of Materia Medica.

PROF. E. QUINE has resigned the chair of Practice of Medicine.

CHICAGO MEDICAL COLLEGE.—University-Day was celebrated by students of the Northwestern University on Monday, February 22. The Chicago Medical College, the Medical Department of the University, was well represented by a delegation of 80 students.

At the general meeting, during the afternoon, brief addresses were made by PROF. S. J. JONES, M.D., representing his colleagues of the Faculty of the Medical Department, and by the HON. HENRY BOOTH, LL.D., DEAN of the Law Department of the University, representing his colleagues of that department.

